# Integrative Model of Human-Animal Interactions: A One Health–One Welfare Systemic Approach to Studying HAI

**DOI:** 10.3389/fvets.2022.656833

**Published:** 2022-07-29

**Authors:** Charlène Leconstant, Elisabeth Spitz

**Affiliations:** Université de Lorraine, UR 4360 APEMAC, Metz, France

**Keywords:** human-animal interactions (HAI), affects, behavior, emotions, affective neuroscience, animal welfare, primary emotional systems, feeling of safety

## Abstract

The Integrative Model of Human-Animal Interactions (IMHAI) described herewith provides a conceptual framework for the study of interspecies interactions and aims to model the primary emotional processes involved in human-animal interactions. This model was developed from theoretical inputs from three fundamental disciplines for understanding interspecies interactions: neuroscience, psychology and ethology, with the objective of providing a transdisciplinary approach on which field professionals and researchers can build and collaborate. Seminal works in affective neuroscience offer a common basis between humans and animals and, as such, can be applied to the study of interspecies interactions from a One Health-One Welfare perspective. On the one hand, Jaak Panksepp's research revealed that primary/basic emotions originate in the deep subcortical regions of the brain and are shared by all mammals, including humans. On the other hand, several works in the field of neuroscience show that the basic physiological state is largely determined by the perception of safety. Thus, emotional expression reflects the state of an individual's permanent adaptation to ever-changing environmental demands. Based on this evidence and over 5 years of action research using grounded theory, alternating between research and practice, the IMHAI proposes a systemic approach to the study of primary-process emotional affects during interspecies social interactions, through the processes of emotional transfer, embodied communication and interactive emotional regulation. IMHAI aims to generate new hypotheses and predictions on affective behavior and interspecies communication. Application of such a model should promote risk prevention and the establishment of positive links between humans and animals thereby contributing to their respective wellbeing.

## Introduction

The study of human-animal interactions (HAI), relationships (HAR) and bonds (HAB) between humans and animals is an increasingly topical issue in many fields of research. Most HAI studies are conducted in the context of research on companion or agricultural animals, along with an increase in recent years of studies on laboratory, zoo and wild animals (1). HAI can be defined as a dynamic process in which previous human-animal interactions form the basis of a relationship that then exerts a feedback effect on the nature and perception of future interactions (2). The study of HAI is an interdisciplinary research area aimed at quantifying the bidirectional effects of the human-animal relationship on human and animal health and wellbeing (3).

HAI have been shown to provide health benefits for humans (4–7), and research also demonstrates that the amount and quality or nature of interactions between animals and their caretakers and handlers have a significant effect on the behavior, physiology and wellbeing of farmed (8–10) and domestic animals (11, 12).

However, in the literature and to our knowledge, there has yet to be a unified theoretical framework allowing to model the way humans and animals interact together. Without the development of an integrative theoretical framework to approach the study of HAI, scientific progress in the various areas of HAI/HAB/HAR will remain limited. In this article, we present the Integrative Model of Human-Animal Interactions (IMHAI) which mobilizes current knowledge from three disciplines: neurosciences, psychology and ethology, as well as processes identified directly from over 5 years of action research in order to propose a conceptual framework that can serve as a common basis for future interdisciplinary work in the field of HAI, the underpinning of which is outlined in section The Integrative Model of Human–Animal Interactions. More specifically, we propose to focus on the study of a process common to humans and animals which we hypothesize is central and conditions HAI: emotions.

Emotions are considered at the heart of many definitions of animal welfare circulating in the scientific community (13–18). Indeed, if we consider that, in animals, emotion has a valence (positive or negative), i.e., that it is pleasant or unpleasant, it actually improves or compromises the animal's wellbeing (19). To date, emotions have mostly been studied in animal welfare science as intra-individual phenomena, i.e., as coordination mechanisms that guide the animal to take appropriate actions (19). Influenced by previous research in humans, several theoretical frameworks have been developed to study and assess emotions in animals, such as the cognitive appraisal theory (20), or the theoretical framework developed by Mendl et al. (21) integrating both the *dimensional approach* to emotions with the *discrete emotions approach*. However, on many occasions, emotions are generated in social contexts: emotions have an important social dimension, have high communicative value, carry meaning (22), and have many impacts on cognitive and social processes (23, 24). In particular, in social species, interactions with peers are critically important to the development of individuals and have a preponderant impact on their health and wellbeing (25, 26). Beyond context, it is therefore highly likely that the emotional regulation of social beings is dependent on the interactions and relationships they have with each other (27).

Based on the previously cited work but also societal developments, there appears to be a concomitant paradigm shift in our current appreciation of animal welfare and human-animal relationships (28). A good example of this is the evolution of the 2020 Five Domains model (29) referencing contemporary verified scientific thinking for assessing animal welfare. This model includes five domains: (1) nutrition, (2) physical environment, (3) health, (4) behavioral interactions, and (5) mental/emotional state, which is the subjective experience of the animal derived from the previous four domains. The notable evolution of the model is in domain 4, previously called “Behavior,” renamed “Behavioral Interactions,” and subdivided according to the nature of the animals' interactions with (a) their environment, (b) other non-human animals, and (c) humans. It appears that the assessment of welfare, previously based on linear causality, has been abandoned in favor of an interactionist approach that studies dynamic and relational phenomena in constant evolution (30).

Through this article, we will present the Integrative Model of Human-Animal Interactions (IMHAI), which argues that attention to emotions during HAI should enhance our understanding of interspecies communication and may open new avenues for positively influencing human and animal health and wellbeing when interacting with each other. In particular, we propose to embed this model in a systemic approach in congruence with the One Health–One Welfare concepts for the study of HAI.

## The Integrative Model of Human-Animal Interactions

The goal of the Integrative Model of Human-Animal Interactions (IMHAI) is to propose a common model for both humans and animals in order to better understand interspecies interactions. We propose to study the primary emotions, according to the individual's perception of the environment as well as to social context. To develop the IMHAI, an inductive qualitative research methodology was used, in the tradition of Grounded Theory (31) and Action Research (32) which propose to theorize different phenomena based on a constant back and forth between research and practice. This methodology enabled us to explore the primary emotional processes occurring in HAI and to gain new theoretical insights. In this analysis, we present the result of a theoretical reflection carried out within the setting of a more global research conducted over a period of 5 years, the results of which are currently being published. At present, the model is intended for the study of interactions between humans and other mammals and birds. Herein, the focus is on HAI/HAR by referring to the non-human animal as “animal” for simplicity of text. However, this model could conceivably be the object of additional modifications and proposals that could allow for the study of interactions between humans and other types of living beings such as fish and reptiles.

### Toward a Systemic One Health–One Welfare Model of HAI

The One Health initiative takes a multidisciplinary approach to optimizing human, animal and environmental health (33), while the One Welfare approach aims to integrate animal welfare, human welfare and the physical and social environment, with the goal of improving global wellbeing and achieving gains in development (34). According to Jordan and Lem (35), “One Health initiatives have traditionally focused on threats to human and animal health, such as zoonoses and a secure food supply, they have not typically promoted an understanding of the many beneficial physical and psychosocial impacts of human-animal relationships and how these can be leveraged to improve both human and animal health around the world.”

New thinking is emerging, particularly in the field of animal-assisted therapy, for the development of a One Health–One Welfare framework applied to HAI (36). The main tenet being that HAI themselves are leveraged for the development of synergistic health and welfare benefits for both the human and the animal during the intervention. To achieve this outcome, Hediger et al. (37) support the need for a systemic approach to studying the interrelationship and reciprocal influence of human-animal relationships by including environmental factors and social contexts. Furthermore, Menna et al. (38) suggest studying HAI as a complex system (39), strongly encouraging the study of an interspecies relationship as a reciprocity. Indeed, in its very etymology, interaction suggests the precept of mutual, reciprocal action of several elements. The notion of reciprocity implies one of feedback, a circular process in which the receiver's reaction to a message (or action) is driven in the direction of the sender and in turn acts as a stimulus, an influence on him within a series of exchanges (40).

Based on these elements, the IMHAI proposes to study HAI from an interactionist and systemic perspective, studying the processes that modulate HAI, taking into account context and environmental factors, in order to promote the establishment of positive interactions between humans and animals that contribute to their health and wellbeing. Adopting an interactionist approach to interspecies social phenomena signifies taking a point of view where the basic unit of social analysis is not the individual action, but rather the system formed by the set of actions that occur between individuals which, in a given context, respond to each other to generate a situation, a reality to be observed and analyzed (40). Finally, it focuses on the communication process considered as a global phenomenon and integrating all patterns of behavior that can have a communicative value, such as speech, facial expressions, gaze, gestures, interpersonal distance, etc.

In the following section Processes Possibly Involved in Interspecies Communication, we propose to explore interspecies emotional communication through the hypothesis of emotional transfer including automatic processes such as emotional contagion but also more complex processes involving social cognition.

### Processes Possibly Involved in Interspecies Communication

#### Emotional Contagion

Emotional contagion, a process considered as the basis of primary empathy, occurs when the perception of another individual's emotional state automatically activates shared representations, eliciting a corresponding emotional state in the observer (41–43). For example, a signal emitted by an individual may induce the same arousal level (i.e., contagion of emotional arousal) and the same valence (i.e., contagion of emotional valence) in the receiver (44). The most recent empirical works have predominantly focused on fright given that the behavioral responses generated by this emotion are easily conditionable and observable under experimental conditions (45, 46).

Primary empathy is a bottom-up control process presumed to be already present in birds and mammals, giving individuals the intrinsic abilities to resonate with the pains and joys of their conspecifics through primitive emotional contagion (42, 47). In a study by Reimert (48), the authors examined the phenomenon of emotional contagion in pigs (i.e., *Sus scrofa domesticus*) and showed that a negative treatment of social isolation not only affected the emotional state of the animal itself, but also that of other pigs that were not subjected to the negative treatment, with the stress effect being prolonged even beyond the duration of the experiment.

The process of emotional contagion is equally studied in the context of interspecies interactions, particularly through the effect of mimicry (body and facial expression) (49). However, motor mimicry is not a prerequisite for emotional contagion, but rather one of its modalities. For example, in juvenile common ravens (i.e., *Corvus corax*), emotional contagion in the context of play could occur through a general mood state transfer resulting in increased locomotion and social play rather than through the reproduction of the same motor patterns (50). Human body odors produced in states of happiness and fear also stimulate animals emotionally and induce sympathetic and parasympathetic changes, suggesting an interspecies transfer of emotions through this means (i.e., autonomic mimicry) (51–53). Emotional contagion through vocalizations is also studied in different animal species (54, 55) and shows compelling results (56, 57). In particular, it is established that animals, like humans, have the ability to discriminate vocal expression of emotions (58). Furthermore, vocalizations have the potential to influence the receivers' affective states through direct (e.g., acoustic startle reflex) or indirect effects [e.g., affective learning and learned affect (59)], which could lead to state matching, as discussed in the section Embodied Communication with processes of social affordance and embodied cognition.

#### Embodied Communication

To study interspecies communication, we suggest that the processes of social affordance and embodied cognitions are key investigation points of interest, as they include the contribution of social cognition in an ecological approach to behavior, where perception is considered as a process emerging from the individual-environment system. According to Gibson (60), both humans and animals are responsive to “affordances,” i.e., to possibilities for action offered by an environment, and are selectively responsive to a specific affordance over another. Social affordances are a subcategory of affordances, namely possibilities for social interaction offered by an environment. In this perspective, a number of different authors [see for example (61, 62)] postulate that the intentionality of actions and the ability to understand the intentions of others are based more on primary and sensorimotor processes than on highly specialized cognitive abilities such as inferences and mentalization. For instance, in a situation where one individual observes what the other is doing, the process of resonance *via* mirror neurons can be understood as part of an intersubjective perceptual process (63). By observing the other individual's expressive actions and movements, in the context of the surrounding external environment, the individual perceives their meaning (61). Thus, perceiving the embodied behavior in the situation provides sufficient information for understanding, reacting and interacting with the other. The ability of humans and animals to communicate with each other therefore does not presuppose an *a priori* cognitive processing for the interpretation of “emotional” signals (64). Emotional expressions (generated by the activation of primary emotional systems) are considered as social affordances produced by individual A to act on individual B (65). In this sense, they precondition individual B for action, depending on the nature of the expressed emotion, the social context, the socio-affective skills and the motor repertoire of the protagonists. By producing emotional expression, individual A instigates different intentions to act on individual B; these intentions to act are not necessarily conscious and can lead to different social consequences. For certain emotional expressions, the aim of the signal from individual A is to make individual B react (anger); for other emotional expressions, the function of the signal is to “contaminate” individual B (i.e., emotional contagion) (fear) by prompting the latter to act in a congruent manner. Other emotions are intended to gain the attention, curiosity and affection of another individual. Thus, in social contexts, emotional expressions provide information about an individual's willingness to enter into a relationship with another individual, or conversely, their unwillingness to enter into a relationship (22). In particular, it would be interesting to study how interspecies emotional transfer influences the characteristics of the interaction and the subsequent relationship, namely the frequency and duration of the interaction, the initiation of physical contact, or even behavioral synchronization and behavioral correspondence (66), that increase mutual affiliation (67).

In the following section Attachment and Interactive Emotional Regulation, we propose focusing on a third process that is likely to influence interspecies communication, namely the interactive emotional regulation associated with the autonomic nervous system, in order to explore the factors potentially involved in the emergence of positive or negative interactions, contributing in the longer term to the establishment of affective links between humans and animals.

#### Attachment and Interactive Emotional Regulation

Through extensive study of attachment and autonomic regulation *via* the vagus nerve, various authors (68–72) argue that the need to connect with others is a primary biological imperative, both for humans and social animals. The findings of their respective seminal works are consistent with the precepts of the attachment theory that were first defined by Bowlby (68, 73). Social bonds strongly support emotional balance and promote wellbeing, mental and physical health throughout the life span of mammals and birds (74–76).

According to Schore (76, 77), the attachment theory is essentially a regulation theory. Interactive emotional regulation implies that emotions are regulated through interaction. It prompts us to depart from the traditional individual-centered analysis of social cognition and proposes to consider social cognition as grounded by neurophysiological processes that are distributed across brains and bodies and is manifested in the coregulation of behaviors (78). In a dyadic interaction, coregulation refers to a process whereby the emotions of both individuals are moderated over time and return to homeostatic levels. Codysregulation, on the other hand, means that the emotions of both individuals are amplified and move away from homeostatic equilibrium (79). For example, through the process of emotional contagion, an interspecies emotional codysregulation can be observed: fear experienced by the human can be transmitted to the animal which, in turn, undergoes a fear-related sympathetic activation, observable at the physiological and behavioral level (80). Repeated experiences of codysregulation lead to difficulties in the establishment of a good relationship, and even to a rupture of bonds (81). In contrast, coregulation promotes emotional security, trust and leads to bonding (82). In this context, both individuals feel a sense of wellbeing and safety that is conducive to the development of emotional bonds and learning. In particular, the interactive emotional coregulation between the child and his caregiver during the first years of life would be linked to the development of a sense of safety and self-regulation skills and would modulate the neurodevelopmental aspect of the child's future adaptive functioning (83–86).

Furthermore, the attachment theory proposes that the history and quality of early interactions with primary caregivers, *via* interactive emotional regulation, shape the young's internal attachment patterns forming more generalized mental representations of self and others as adults (68, 87, 88). Researchers thereafter studied how the human's attachment style can affect the relationship toward the animal (89, 90). For example, dogs (i.e., *Canis lupus familiaris*) which are aggressive toward humans are less sociable, and have owners who are less emotionally stable, more distant, and less clingy and controlling, compared to non-aggressive dogs (91). Avoidant owners are less responsive to the dog's needs and do not provide a secure-base for the dog when needed, which would result in a higher risk for the dog to develop a separation-related disorder (92). Domestic animals are also likely to show attachment patterns to their owners (93–95), but these must be empirically identified. Interestingly, there is research in social neuroscience that identifies neural pathways associated with attachment patterns (96) and how dysfunctional attachment patterns impact interpersonal interactions [see (81, 97) for more details]. Indeed, while the ability of domestic animals to provide a safe-haven and secure-base for humans is well documented (98–100), the moderating role of attachment styles of pet owners to the animal is not solely an extension of the general interpersonal attachment patterns that we have identified in humans (101). Additional studies thus appear warranted with regard to specific qualities of the human-animal bond.

In any event, studying the autonomic nervous system when individuals interact together could provide valuable insight on how emotion regulation impacts interspecies relationships. The model of neurovisceral integration (102) postulates that cardiac vagal tone, indexed by heart rate variability (HRV), can indicate the functional integrity of neural networks involved in emotion-cognition interactions. For example, in humans, it has been shown that lower resting HRV is associated with hyper-vigilance and maladaptive cognitive responses to emotional stimuli, thereby impeding emotional regulation (103, 104). Therefore, measuring the autonomic state *via* HRV is likely to provide insight (1) on the individual's emotional regulation capacity, and (2) on how the autonomic state of interacting individuals evolves according to the expressed emotional states creating coregulation or, on the contrary, codysregulation. Note that the synchronization of heart rates is an appealing notion indicating shared autonomic experiences between individuals; however, the synchronization of heart rates between animals and humans has not been definitively proven (105).

Thus, beyond taking into account the expressed affect, it is also a matter of taking into account the capacities of each individual for emotional regulation. In this sense, actions aimed at preventing risks in HAI do not only consist in preventing stimuli that could frighten an animal or human, but also in increasing their respective capacity to regulate their emotions in the presence of various stimuli. In addition, the goal is to study the manner in which an individual is able to regulate his emotions alone (self-regulation) as well as during an interaction with others (interactive emotional regulation). In particular, in the setting of interspecies interactions, interactive emotional regulation could require considerable adjustments on the part of both humans and animals, justifying a thorough investigation of this process in future research.

Throughout the above section Processes Possibly Involved in Interspecies Communication, a central element appears to stand out through the process of interspecies communication: emotions. The latter could represent the basic unit contained in the “messages” exchanged between individuals, perceived in a conscious manner or not. The emotions could take different channels—olfactory, visual, auditory—allowing their transmission to the other individual and produce different effects on the receiver, preparing him to interact in a phenomenon of reciprocity. In the following section Emotions as a Key Process for the Study of HAI, we propose to detail emotions as a key process for the study of HAI, and in particular the basic or primary emotions that we consider fundamental for designing a model of HAI in a One Health–One Welfare systemic approach.

### Emotions as a Key Process for the Study of HAI

Despite an abundance of literature both in biology and psychology on the science of emotion, its definition remains the subject of passionate debate and divergent views. Indeed, emotion as a psychological entity or as a category with clearly-defined boundaries does not exist. The concept of emotion covers a myriad of phenomena involving a diverse array of activation processes and configurations (106). Most taxonomies of human emotions, in agreement with previous models [i.e., (107, 108)], postulate the existence of a fairly small set of basic emotions such as sadness, joy, anger, fear, surprise and disgust (109). The emotions described in these taxonomies are not only recognizable in human faces (110, 111) but also in those of animals such as different primate species (112–115), the dog [DogFACS: (116)], the cat [Cat FACS: (117)], or the horse [EquiFACS: (118)]. Thus, there appears to be evolutionary continuity between humans and mammals and birds in the recognition of emotional states (119, 120).

The ability of all animals to experience affective states was the subject of an international consensus at the Francis Crick Memorial Conference on Consciousness in Human and non-Human Animals, recognizing that: “The absence of a neocortex does not appear to preclude an organism from experiencing affective states. Convergent evidence indicates that non-human animals have the neuroanatomical, neurochemical, and neurophysiological substrates of conscious states along with the capacity to exhibit intentional behaviors.” (121). In this sense, primary emotions meet the requirement of the “One Biology” concept defined by Tarazona et al. (122), which adjoins and complements those of One Health–One Welfare, implying that biological principles are the same for humans and all other animals, although there are specific differences between species and individuals. Because humans and animals share similar primary emotional states that are recognizable across species, we hypothesize that it is possible to study HAI on the basis of primary emotional states that influence interspecies communication. In particular, we will focus on primary/basic emotions, emotions that are studied with reference to central emotional states, evolutionarily conserved, whose brain activation is identifiable in the brains of a wide variety of animal species (123).

Currently, research remains to be conducted to determine the nature of these central emotional states, some of which, such as fear, could be common to the entire animal kingdom (124–126). While awaiting new results in this field and an eventual broad consensus, we have chosen to study the basic or primary emotions common to humans and mammals (and birds), identified and documented in the seminal work of Jaak Panksepp, of which we propose to expose the key elements with, in particular, the description of the seven primary emotional systems, in section Description of the Seven Primary/Basic Emotional Systems. The study of primary emotional systems was first carried out from a comparative perspective between humans and animals, and in this article, we propose to use the latter for the study of interspecies interactions. However, certain authors may consider emotions other than those presented in this article, provided they are identified in both humans and animals.

#### Description of the Seven Primary/Basic Emotional Systems

According to Panksepp (127–129), much of human and animal behavior depends on specific emotional circuits in the brain. These connections not only help organize coherent behavioral responses to important environment challenges, but also provide emotional values to guide everyday decision-making. Primary-process emotional feelings are organized within primitive subcortical regions of the brain including the basal forebrain, diencephalon, midbrain and other brainstem systems, that are anatomically, neurochemically and functionally homologous in all mammals studied^1^ (129, 131). Mammalian brains contain at least seven basic emotional systems: SEEKING, PANIC, CARE, PLAY, LUST, FEAR and RAGE (capital letters reflect a proposed primary-process terminology by Panksepp, so as to minimize semantic confusions). The anatomical trajectories of these seven higher emotion-generating subcortical systems are wired into the neocortical limbic regions of the brain (43). The primary emotional systems that give rise to emotional states involve various components: neurodynamics (excitation and inhibition), neuromodulators (neurochemicals) as well as neuroanatomical (brain structures and areas) and behavioral aspects. Detailed descriptions of these systems are provided below, as outlined by Panksepp (43, 132, 133), including their primary functions, key brain areas (anatomies), neuromodulators and predetermined key behaviors associated with these systems (see Table 1).

**Table 1:** Description of the seven primary emotional systems identified by Panksepp (71).

	**Key brain areas**	**Key neuromodulators**	**Key behaviors**
**SEEKING**			
Activation of the seeking and desire system in the brain is associated with contact and engagement with the environment; it fosters the individual's curiosity and the appetite to explore and discover. This desire to move forward in the environment is essential for individuals in order to find the resources and partners necessary for their survival. This appetitive motivational system (assimilated with the “reward circuit”) produces an eager anticipation of forthcoming resources when conditioned. It maintains connections with all other emotional systems and allows them to function effectively^a^	Ventral tegmental area (VTA), medial forebrain bundle (MFB), nucleus accumbens (NAcc), medial prefrontal cortex (mPFC), mesolimbic and mesocortical outputs, lateral hypothalamus, periaqueductal gray (PAG)	Dopamine (+), glutamate (+), opioids (+), neurotensin (+), orexin (+), many other neuropeptides	Sniffing, active exploration of the environment or an object
**PANIC**			
Activation of the “separation distress” system in the brain motivates the individual to seek out connections with others that provide a feeling of safety. From birth, young mammals and birds express distress vocalizations that resemble panic attacks when isolated; reuniting with their caregiver promotes social bonding	Anterior cingulate, bed nucleus of the stria terminalis (BNST) and preoptic area, dorsomedial thalamus, dorsal PAG	Opioids (–), oxytocin (–), prolactin (–), corticotrophin releasing factor (CRF) (+), glutamate (+)	Distress vocalizations, clawing (in some species), active search for a congener
**CARE**			
Activation of the caregiving system in the brain prompts the individual to respond to the search for attachment of others, through tender and loving acts. Brain evolution has provided safeguards to ensure that parents take care of their offspring. The CARE system generates incentives for the parent to nurture and provide emotional and physical care to its young in order to bond emotionally and provide a sense of safety to the offspring^b^	Corticomedial amygdala, anterior cingulate, BNST, VTA, MFB, medial hypothalamus and preoptic area, ventral PAG	Oxytocin (+), prolactin (+), dopamine (+), opioids (+/–)	Offspring care behavior: feeding, warmth, affectionate physical contact, holding, incubating Affiliative behaviors, grooming.
**PLAY**	
Activation of the play system in the brain strengthens social learning, skills and connection with others. It is a vector of hedonic sensations. The young have a strong desire for very communicative physical play, through which they learn the affective values of social interactions	VTA, dorso-medial diencephalon, parafasicular thalamus, PAG, mPFC	Opioids (+/–), glutamate (+), acetylcholine (Ach) (+), endocannabionoids, and probably many other neuropeptides	Rough-and-tumble play
**LUST**	
The primary function of the sexual desire system is to perpetuate the species. Activation of the LUST system in the brain activates seductive behaviors and the sexual act. Male and female sex drives are mediated by several distinct brain neuropeptide circuits, the activities of which are regulated by their respective gonadal steroids	Cortico-medial and lateral amygdala, BNST, preoptic hypothalamus, ventromedial nucleus of the hypothalamus (VMH), ventral medial forebrain bundle (vMFB), ventral and dorsal PAG, PFC.	Steroids (+), oxytocin, vasopressin, luteinizing hormone-releasing hormone (LH-RH), cholecystokinin (CCK)	Seduction behaviors and the sexual act
**RAGE**	
Activation of the anger system in the brain is triggered by frustration and attempts to curtail an individual's freedom of action. The activation of the RAGE system generates defensive mobilization behaviors (fight) and elicits the neural activation of the FEAR system in the opponent. This system is also activated in social situations involving social hierarchy (dominance/submission), competition for access to resources, and overcoming obstacles	PFC, ventral MFB, medial amygdala to BNST. Medial and glutamate (+) perifornical hypothalamic to dorsal PAG (dPAG)	Substance P (+), neuropeptide Y (NPY), Ach (+), glutamate (+)	Defensive mobilization (fight), bite
**FEAR**	
The fear system contains genetically encoded action schemes aimed at optimizing the safeguard of the individual (protect against predators, dangers, risks of injury and premature death) and reduce the likelihood of being exposed to pain. Animals exhibit flight or freeze behavioral responses	Central and lateral amygdala to medial hypothalamus and dorsal/ventral PAG; vMFB, PFC	Glutamate (+), diazepam binding inhibitor (DBI), CRF, CCK, alpha*-*melanocyte stimulating hormone (alpha-MSH), NPY	Flight or freeze

^a^*For example, a relevant model of neurobiological regulation of affiliation in mammals (74) suggests that dopamine plays an important role in incentive–reward motivational processes associated with the appetitive phase of affiliation (SEEKING system), that endogenous opioids are involved in the consummatory phase of socialization (i.e., CARE and PLAY system), and that oxytocin and vasopressin enhance the perception and memory of affiliative stimuli (134)*.

^b^*In the current state of research, the CARE circuit has mainly been studied in females, in particular maternal behaviors with the action of peripheral estrogen, progesterone, prolactin and cerebral oxytocin. Males would appear to have an inherently weaker CARE system, and therefore would require further emotional education to become fully engaged caregivers (71)*.

The activation of emotional systems emerges from the synthesis of an individual's perception of the environmental interactions that involve the individual personally, which include not only challenges and threats but also the ability to respond to them (22, 135). Primary emotional systems play an important role in the expression of emotions in humans and animals, whether or not they have a subjective perception of these states, and mediate what is commonly referred to as “action tendencies” (136). These systems organize physiological and behavioral responses to prepare the body for an optimal response, creating very broad variations in the responses observed. Thus, emotional expressions reflect the state of an individual's permanent adaptation to constantly changing environmental demands. When primary emotional systems function properly, they promote flexible adaptation to environmental changes (137).

The seven primary emotional systems do not imply that emotional states are limited to seven emotions. Each system has abundant descending and ascending components working together to coordinate various instinctual emotional behaviors and associated autonomic changes, as well as raw affective states (as evaluated by the rewarding and punishing properties of these systems) (129). These primary emotional systems interact with each other and form a basis from which an infinite number of emotional states and expressions can emerge, becoming increasingly complex through secondary processes (learning and memory) and tertiary processes (higher-order functions of the mind associated with cortical expansions enabling thoughts and planning) (138, 139). On the other hand, the perception and processing of information related to primary, secondary and tertiary processes can vary not only from one species to another, but also at the individual level. For instance, in adulthood, the activation of the CARE system, which mediates grooming behaviors in social species, will primarily be activated to provide care to the offspring in so-called solitary species.

The primary emotional systems are distinct and represent a primary affect dedicated to a specific adaptive function. However, they are also highly interconnected with each other. A systemic study of the interconnections between primary emotional systems could be very rewarding in the field of HAI. Indeed, although it is complex to study the interconnection between an individual's emotional systems in real time, we suggest that their study can support metapsychological thinking regarding behavior and generate hypotheses that are important for the development of new study designs.

First, some emotional systems function antagonistically; that is, they activate and inhibit each other, producing bidirectional effects on behavior. When the defensive systems RAGE and FEAR and the separation distress system PANIC are activated, they inhibit the exploration system SEEKING and the social systems CARE, PLAY and LUST in the individual (71, 129). The RAGE, FEAR and PANIC systems can thus be associated, more generally, with negative affects, whereas the SEEKING, CARE, PLAY and LUST systems are associated with positive affects (140).

Second, there are strong interconnections between certain emotional systems. For example, we can cite the existing interconnections between the triad of emotional systems PANIC, CARE and SEEKING, which have already been described at length in the literature by various authors (68, 73, 141–143). On the one hand, in social species, the crying of the infant (PANIC system activated) activates the parent's PANIC separation distress system, which in turn activates the parent's caregiving system (CARE system). The PANIC system enables the empathy function in this instance, that of being sensitive to the suffering of the other, and the CARE system enables the response with tender and caring acts (43). The strong interconnection of the PANIC and CARE emotional systems allows the parent and the infant to enter into an emotional connection, contributing to the child's good physical and emotional development. This internal dynamic appears from our point of view as extremely rich in learning, in particular because it conditions an appropriate response during an interaction as well as an emotional connection between two individuals: here, the parent comes to console the infant, to feed him, etc. On the other hand, the separation distress system (PANIC system) and the exploration system (SEEKING system) maintain an important interconnection, where the activation of one inhibits that of the other (144). Indeed, rewarding social interactions cause a strong secretion of endogenous opioids (inhibited PANIC system), which in turn act on the mesolimbic dopamine pathway by stimulating dopamine production in ventral tegmental area neurons, activating the SEEKING system, which generates a state of arousal and motivation to explore the environment and undertake actions (145, 146). As a result, the more the individual feels reassured by the presence of significant others, the more likely he will actively explore the environment (147).

Thus, rather than studying the expression of a single emotional system, we suggest that it is necessary to look at the interconnections between the primary emotional systems of interacting individuals, constituting a complex system. In the following section The PLAY System Involved in Social and Emotional Regulation, we present in more detail the PLAY emotional system, which is of particular interest for the study of HAI, since this system is essentially activated in the context of social interactions and participates in their regulation.

#### The PLAY System Involved in Social and Emotional Regulation

Social play is a window on cognitive and communicative abilities of species (148, 149). The PLAY system is thought to provide fundamental learning experiences of emotional coregulation leading to higher forms of empathy. A key function of the social PLAY system is to help young animals develop social skills and refine the subtle social interactions needed to thrive (132). In this manner, the PLAY system may be one of the major emotional forces driving the epigenetic construction of higher social brain functions, perhaps even of mirror neurons (150). It may also be the main primary brain process favoring numerous types of social communication (151).

Interspecific play in particular is a fertile venue to explore the capacity to correctly perceive and interpret signals emitted by partners (148). Porges (152) defines interactive play as a neural exercise which requires synchronous and reciprocal behaviors between individuals as well as an awareness of the level of social engagement of each individual. When the PLAY system is activated, play sequences can also activate predetermined action patterns of the emotional systems RAGE and FEAR, sometimes LUST system, without threat or intent to harm (71). The PLAY system appears to be the only emotional system in which mobilization of the body is both activated and inhibited. Through rough-and-tumble play, such as mimicking fights, young mammals would not only learn behavioral patterns that enable them to learn how to flee or defend themselves, but also emotional self-regulation skills (153, 154). Acquisition of these skills would effectively inhibit defensive responses that are inappropriate to the situation. The development of self-regulation would occur through interactive regulation, constructed in settings of dyadic interactions (155). However, given that the PLAY system has strong neural connections with both the FEAR and RAGE systems (71), the animal can easily switch from a play behavior to behavior that poses a risk of injury to humans.

Researchers now consider that play has both immediate and delayed benefits (156, 157). However, the benefits associated with play do not make it a reliable indicator of the presence of a state of wellbeing in the individual. First, in adults, activation of the PLAY system is less intense than in youths. Second, studies in New World monkeys, i.e., *Callithrix jacchus*, and horses, i.e., *Equus callabus*, have shown that adults who play the most have the highest stress score (158, 159). Activation of the PLAY system in adults could be used as a coping strategy with the function of releasing aggressive tensions while preserving social bonds, thus promoting emotional resilience (160). The PLAY system could also enhance the individual's ability to be flexible in using different coping strategies according to the characteristics of the situation (161, 162), by enhancing ANS flexibility in humans and animals (163).

In the following section Framework for the Observation and Assessment of Primary Emotions Across Species, we propose a reflection regarding the development of a framework for the observation and assessment of primary emotions during HAI, drawing on a holistic and interdisciplinary approach.

#### Framework for the Observation and Assessment of Primary Emotions Across Species

**Figure 1:**
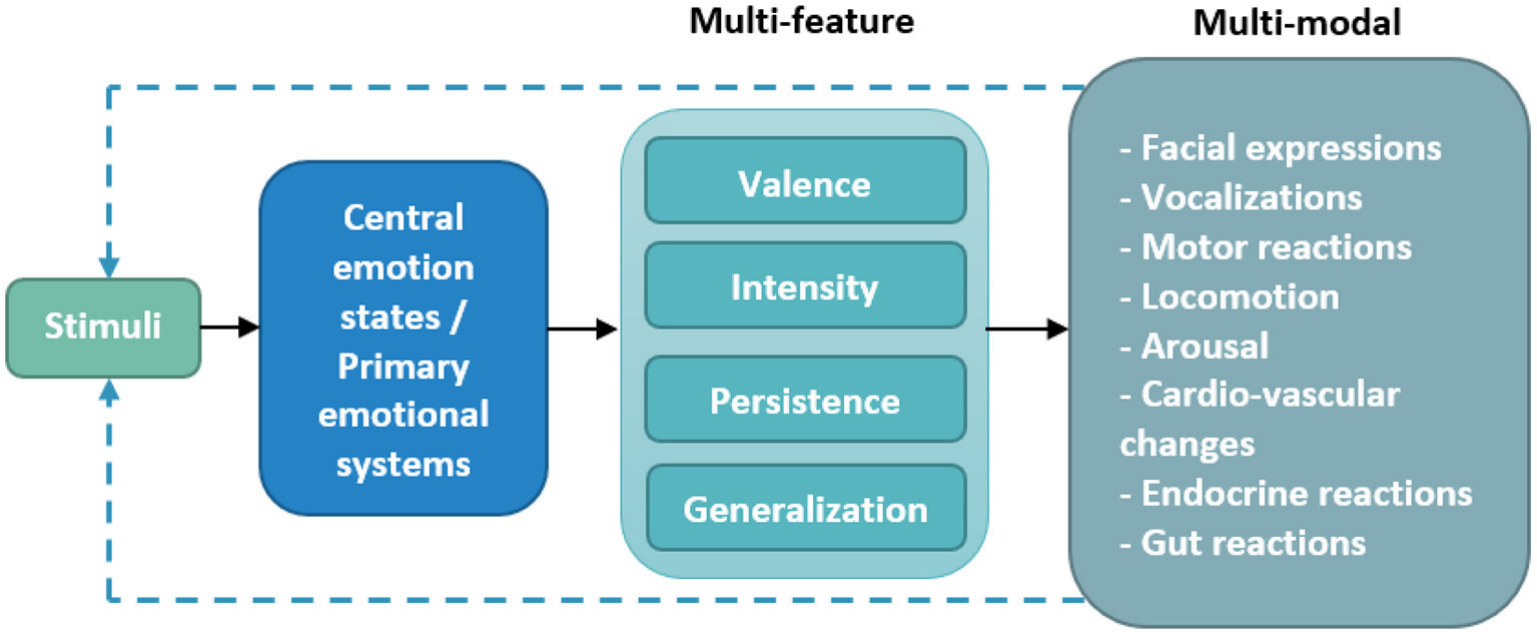
Expression of emotions across species.

To assess the activation of central emotional states or primary emotional systems, it is necessary to develop various methodologies depending on the observed species and the surrounding context. Indeed, in order to be able to study the circularity process inherent to HAI, i.e., the effect that the sender's message or action has on the receiver, and then the impact of the receiver's reaction on the sender, we must be able to identify in the individual, the activation of primary emotional systems and understand their impact on the physiological and behavioral responses observed. Following the principle of One Biology, primary emotions provide a common framework for the study of human and animal behaviors, allowing observations to be made using the same measurement tools. As such, we suggest that the primitive emotion framework put forward by Anderson and Adolphs (123) is relevant for the study and assessment of emotions. The basic emotion features proposed by Anderson and Adolphs (123) (see Figure 1) include (1) valence (emotions are positive or negative), (2) persistence (emotions tend to outlive their trigger), (3) intensity (emotions can be weak or strong), and (4) generalization (the same emotion can occur in different contexts or be triggered by different stimulus conditions). For these authors, all behaviors of living beings, from humans down to the most primitive organism, must fulfill these four characteristics in order to be considered as the expression of an emotion. Figure 1 illustrates the different characteristics and modalities through which emotions are expressed, forming a prism from which it is possible to observe and assess the expression of emotions.

The assessment of interspecies emotions can be studied either directly in the brain, and/or through different listed modalities. Central emotion states (i.e., Panksepp's primary emotional systems) trigger “action systems” in multiple modalities, notably behavioral, physiological and biochemical, offering numerous possibilities to measure the activation of the primary emotional systems in the brain in an indirect and non-invasive manner. For example, the emotional state of fear can cause eye widening, increased breathing, attentional changes, blood redistribution, hormone release, motor responses such as tonic immobilization or flight, and sustained avoidance (164); another example is activation of the neural circuits associated with the SEEKING system often leads to an increase in approach and exploration locomotor behaviors, scent behaviors, and orientation of gaze and ears toward the object. The behavioral observation of the activation of primary emotional systems can be conducted from the ethogram specific to the observed species.

Moreover, the study of primary emotional systems in interspecies interactions poses even greater challenges. A rigorous holistic view of interspecies emotional communication remains to be established through an interdisciplinary approach including field professionals and researchers from various backgrounds, in order to more extensively explore the biological and behavioral bases of the human-animal emotional relationship. For Scopa et al. (44), one of the keys to accessing the emotional interspecies exchange would be to consider the whole of the autonomic emotional responses with a multimodal, multisensory and multidimensional analysis.

The study of HAI by evaluating the activation of primary emotional systems could eventually allow determining the valence of human-animal interaction but also the function it serves, with the consequences that this interaction produces in terms of wellbeing. Rault et al. (165) propose a series of indicators that allow a holistic analysis of HAI in order to determine the positive valence of the interaction, such as the movement and location of the animal in relation to the human, its expressive behavior, the attitude of the animal toward the human, and the characteristics of the interaction during and after (i.e., initiation of physical contact, frequency or duration of physical contact, gaze, etc.). The authors recommend caution in assessing a positive HAI since, for example, the animal's motivation to interact with humans may be in conflict with other motivations at the time, while some indicators of a positive HAI may be species-specific.

Hence, it is necessary to investigate various methods of biological, physiological, neurological, cognitive and behavioral measurements (166–168) in order to characterize valid markers of emotional processes during HAI. However, even before investigating these questions through new empirical research, the eventual undertaking of large-scale meta-analyses could provide a major contribution to the field of HAI, since it would allow cataloging the evidence previously obtained in different disciplines and provide insight into phenomena that remain to be studied.

In the following section The Perception of Safety, an Essential Process for the Study of HAI, we propose to add a complementary process to the study of HAI—the feeling of safety—which we hypothesize modulates the activation of primary emotional systems. Its evaluation would then allow anticipating the behavior of humans and animals in order to prevent risky situations and favor positive interactions.

### The Perception of Safety, an Essential Process for the Study of HAI

The concept of safety encompasses the notions of physical and psychological security. It is a perceived safety, which may be more or less removed from the reality of the situation. Feeling safe is recognized as a central component of mental health, wellbeing and post-traumatic growth (72, 169). Conversely, feeling unsafe has been identified as a potentially huge source of distress due to its negative effects on life satisfaction and wellbeing (170, 171). Living in a safe environment is essential for personal and social development and helps predict higher levels of satisfaction with life as a whole (172).

Safety cues alert the organism to when the environment is safe, thereby promoting behaviors that lead to natural rewards, such as feeding and mating, whereas danger cues inhibit these behaviors. Thus, safety signals possess reinforcing properties, allowing the individual to learn to be safe. At the neural level, it has been shown that a safety signal can not only inhibit the output of the amygdala complex, but also reduce the ability of a sensory cue to excite the lateral amygdala (173). As a result, the individual who perceives safety cues will be less likely to be frightened in response to different stimuli. More than the absence of danger, the safety stimulus is associated with the presence of protection from danger. In mice, its presence leads to increased exploration and appetitive behaviors (173, 174), reduces immobility and anhedonia, and is thought to have anxiolytic and antidepressant effects (173, 175, 176). When safety learning is impaired, it can lead to maladaptive behavior, chronic stress and mental disorders. Indeed, since the feeling of safety is subjective, appropriate discrimination between safe and unsafe situations and the subsequent decrease in the expression of fear in the presence of safety cues becomes decisive to survival and mental health (177).

#### At the Interface of the Environment and the Individual: The Perception of Safety

According to several authors (69, 72, 102), the basic physiological state is co-determined by the perception of safety. This perception of safety directs a mammal or bird to prefer a familiar vs. unfamiliar environment, looking for cues that it is safe from harm (177). According to Porges (178, 179), the feeling of safety arises primarily through a neural process that assesses risk, without awareness and in a reflex manner, based on a variety of signals from the environment and interoceptive stimuli, and triggers changes in the autonomic state to support adaptive responses.

In order for an individual to be socially engaged, i.e., to express positive social behaviors toward others as well as exploratory behaviors, it is necessary for the central nervous system (CNS) to recognize that the subject is in a safe and secure environment by processing sensory information through neural pathways (180). When the individual perceives safety, the individual's defensive mobilization reactions are then inhibited (181). Vagal influences related to the CNS optimally regulate the autonomic state of the body, with the ANS supporting health, growth and restoration. Particularly, the vagus nerve helps slow the heart, inhibits the hypothalamic-pituitary-adrenal (HPA) axis, reduces inflammation, and regulates self-protective defensive reactions to maintain an optimal arousal level within a functional energy zone [e.g., optimal autonomic balance between the sympathetic nervous system (SNS) and the dorsal vagal influences] (152, 182, 183). The activation of the exploration system (SEEKING system) and social systems such as CARE, PLAY and LUST regulated by vagal influences should help the individual to discover his environment with a sense of safety and pleasure and to engage socially with others (184). Moreover, it can be assumed that by moderating SNS activity, vagal influences should facilitate the activation of the FEAR and RAGE systems without hampering the individual's ability to remain socially engaged.

When, on the contrary, the situation is perceived as unsafe, the ventral vagal function supporting social engagement is dampened or withdrawn. The individual is no longer able to engage in social behaviors and positive interactions with others or in exploratory behaviors. The ANS is optimized to support defense, not health (185). Central vasopressinergic pathways (AVP) change the set-point of the baroreceptor reflex in order to facilitate sympathetic excitation and potentiate mobilization behaviors (180). The various defense responses can be classified into two general forms, namely active defense and immobility (186, 187), and are principally mediated by the FEAR and RAGE systems. These defense reactions can manifest themselves in a chain sequence, called “defense cascade”' (188, 189), with a dominance of the SNS or dorsal vagal influences, according to the degree of danger of the situation perceived by the individual (180) (see Table 2).

**Table 2:** Examples of observable behaviors based on three types of autonomic regulation and their consequences for the organism.

**Autonomic state**	**Observable behaviors**	**Consequences on the body**
Activation of the ventral vagus nerve in situations perceived as safe	Exploration of the environment and social engagement: exploration orientation, prosodic voice, positive facial expressions, welcoming gestures, visual and body orientation toward the object, the individual, and/or the location	Short-term effects: ability to create social bonds, ability to function normally without being overwhelmed by stress, good adjustment, promotes reasoning and finding rest Long-term effects: wellbeing, optimization of mental and physical health
SNS activation in situations perceived as unsafe	Defensive orientation, increased muscle tone and tension, restlessness, tonic immobility, fight and flight behaviors	Short-term consequences on the body: acute stress, hypervigilance, irritability, aggressiveness Long-term consequences: chronic stress, psychosomatic illnesses, behavioral disorders
Activation of the dorsal vagus nerve in a perceived life-threatening situation	Disorientation, physical and emotional numbness (slow motor reactions and reactivity, orientation toward the environment and inhibited sensory vigilance), nausea, defecation, fainting, feigning death	Short-term consequences: biobehavioral shutdown, dissociation Long-term consequences: learned helplessness, resignation, chronic dissociation

In addition, beyond the identification of external factors of danger, such as the presence of a predator, we propose that the FEAR system can be activated on the basis of the absence of safety elements, such as for instance the absence of a shelter to hide, but also of internal stimuli pertaining to bodily sensations and the internal state of the body. For example, the interoceptive information of hunger, thirst, pain and fatigue decreases an individual's ability to cope, making the latter more vulnerable to danger, and will tend to express defensive behaviors (aggression, threats, biting, etc.) toward other individuals (69).

By taking into account the process of safety perception, it becomes possible to anticipate the occurrence of behaviors in two main categories: (1) social and exploratory behaviors, and (2) defensive behaviors. When the situation is perceived as safe, the individual will exhibit social and/or exploratory behaviors, which foster the establishment of positive links between individuals and contribute to the formation of lasting relationships that promote wellbeing. On the contrary, when the situation is perceived as dangerous or life-threatening, the individual will exhibit defensive behaviors such as fleeing, fighting or freezing/feigning death, which increase the risk of injuries and accidents during HAI. When these are repeatedly present, without being appeased during the interaction, they contribute to the establishment of deleterious relationships between individuals, with harmful consequences for their health and wellbeing.

#### The Feeling of Safety in Social Species

In social species, the absence of external danger is not sufficient to feel fully safe. Inclusion in a social group is an essential survival strategy, in particular for protection from predation as well as for easier access to resources (190, 191). Thus, the emergence of a sense of safety is directly linked to the perception of a meaningful emotional and social presence (192). In this context, memorization of safety cues is likely to help avoid danger and recognize a supportive conspecific [for review, see (193, 194)], and as such is an important motivation for repeated social contact seeking. In keeping with the attachment theory, we hypothesize that the separation distress system (PANIC system) is involved in proximity maintenance, which refers to a constant, non-conscious assessment of the distance (physical or mental) separating us from an individual to whom we attach importance. Indeed, Panksepp and Panksepp (43) consider that the activated PANIC networks (i.e., separation distress system) probably correspond to a form of mental suffering that evolved from pre-existing systems that mediated the affective qualities of physical pain, specifically the thalamocingulate division of the brain—which includes the cingulate cortex and connected medial thalamic nuclei (195). Thus, activated PANIC networks are vectors of mental suffering (associated with a decrease in endogenous opioid levels), which motivates the individual to seek contact with other individuals (130, 196). For example, it has been shown that during social acceptance, activation of the μ-opioid receptor in the left ventral striatum is positively correlated with an increased desire for social interaction (197). It has thus been proposed that the activation of the PANIC circuit (related to experiencing the emotional pain of social loss) contributes to the development of attachment as a major force that guides the construction of social bonds and reflects feelings of emotional deprivation and separation anxiety (136).

As previously defined by Bowlby (198) with the notion of “secure base,” the reunion with conspecifics creates a calming of the PANIC system and participates in the creation of the feeling of a “secure neurochemical base” within the brain, *via* endogenous opioids and the action of neuromodulators such as oxytocin (71). According to Depue and Morrone-Strupinsky (74), in social species, with the evolution of care and attachment, the oxytocin-opiate system (CARE system) has become a key affect regulation system, whereby there is a co-assembly of different affects such as contentment, feeling of safety and wellbeing, as well as various physiological effects on pain thresholds, the immune system and brain maturation that are partially regulated by these neurohormones (75, 77, 144, 180). Signals and stimuli from CARE system activation, such as stoking, holding, tone of voice, facial expressions, and social support have thus evolved as natural stimuli that inhibit the separation distress system (PANIC system) and have the effect of calming and soothing the receivers (199–201). The oxytocin-opioid system (CARE system) is thus particularly linked to soothing, calming, feeling of safety and social connectedness (74, 202, 203).

In humans, in adulthood, the ability to visualize a mental presence, for example knowing that a loved one will soon be found, may be sufficient to maintain the PANIC system stable (204). The system is then regulated by top-down cognitive processing and through self-regulation skills. In so-called “solitary” species, the sense of safety is not dependent on the presence of other conspecifics (134). It is rather the aspects of their vital domain which constitute the vectors of safety such as their shelter and familiarity with their environment (205, 206). These tangible elements are likely to allow the emergence of a neurochemical basis for safety, also mediated by endogenous opioids (207).

#### Feeling of Safety and At-Risk Prevention Associated With HAI

Understanding the implication of safety perception on the activation of primary emotional systems should foster the prevention of risk situations in HAI. In particular, increasing safety cues in the environment while taking into account the individual's internal signals (pain, fatigue, etc.) should prevent the occurrence of the individual's defensive reactions. The safety signals are to be adapted according to the species, but also to individual factors (age, past experiences, emotional management capacity, etc.). On the basis of the previously mentioned work (see sections Description of the Seven Primary/Basic Emotional Systems and The PLAY System Involved in Social and Emotional Regulation), it is possible to develop the feeling of safety of the animal and the human, both from the elements of the context, but also from the relationship itself, since it appears that the human and the animal have the intrinsic capacity to become a source of safety for each other, as a signifying being or attachment figure.

The identification of the affect associated with the animal's behavior could have many positive consequences in the human-animal relationship. On the one hand, the identification of situations generating panic (PANIC system), fear (FEAR system) and anger (RAGE system), associated with a sympathetic activation, will allow the prevention and a proper response to the activation of these primary emotional systems, and thus reduce incidents and the risk of injury. In addition, the deliberate action of inferring the affect underpinning this behavior could increase the empathy capacities of humans toward animals and allow humans to respond in a manner more adapted to the animal's emotional state, thus strengthening their bonds. Indeed, the capacity for empathy, in both animals and humans, is intended to be a true driver of prosocial behavior, when sensitivity to the distress of others is accompanied by a desire to ensure their wellbeing (208). On the other hand, by promoting the activation of the SEEKING, CARE and PLAY social emotional systems, through an autonomous vagal regulation, it becomes possible to improve the learning and training conditions of animals, in particular through the development of playful learning methods that promote confidence and the experience of hedonic sensations (9). In general, identifying specific emotional states and promoting a sense of safety will improve the management of animals and, consequently, the wellbeing of both the animals and humans.

### Synthesis of the Integrative Model of Human-Animal Interactions

**Figure 2:**
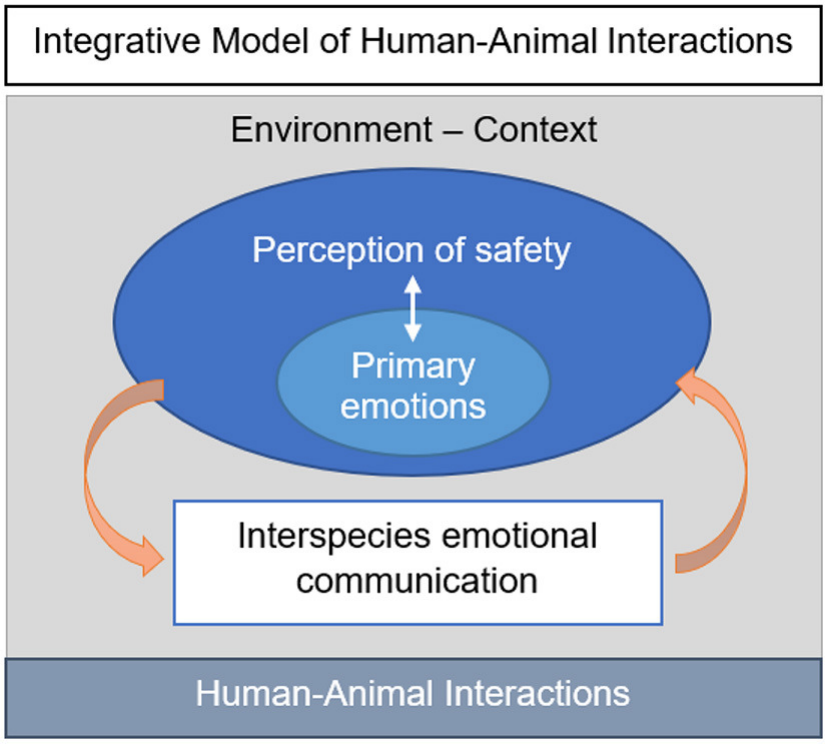
Diagram of the integrative model of human–animal interactions.

The IMHAI proposes to model human-animal interactions by hypothesizing the primary emotional systems that are activated during the interaction. The context and environment in which HAI occurs plays an indirect role on HAI, while the perception of safety modulates the activation or inhibition of primary emotional systems, expressed through the process of interspecies emotional communication, as illustrated in Figure 2.

The IMHAI offers a theoretical framework that can guide us in our observations and the prediction of hypotheses. In practical use, the IMHAI is intended to help both researchers and field professionals relate affect to observed behavior. The modeling of primary emotional systems should allow metareflective activity in the observation of HAI. This modeling can be used to code each observed behavioral sequence, based on video recordings for example, but also allow the possibility of illustrating only selected reactions and behaviors which appear to be the most decisive for understanding HAI. The modeling of HAI must, among other considerations, take into account the stage of evolution of the species studied, its degree of domestication, socialization and familiarity with the other individuals.

**Figure 3:**
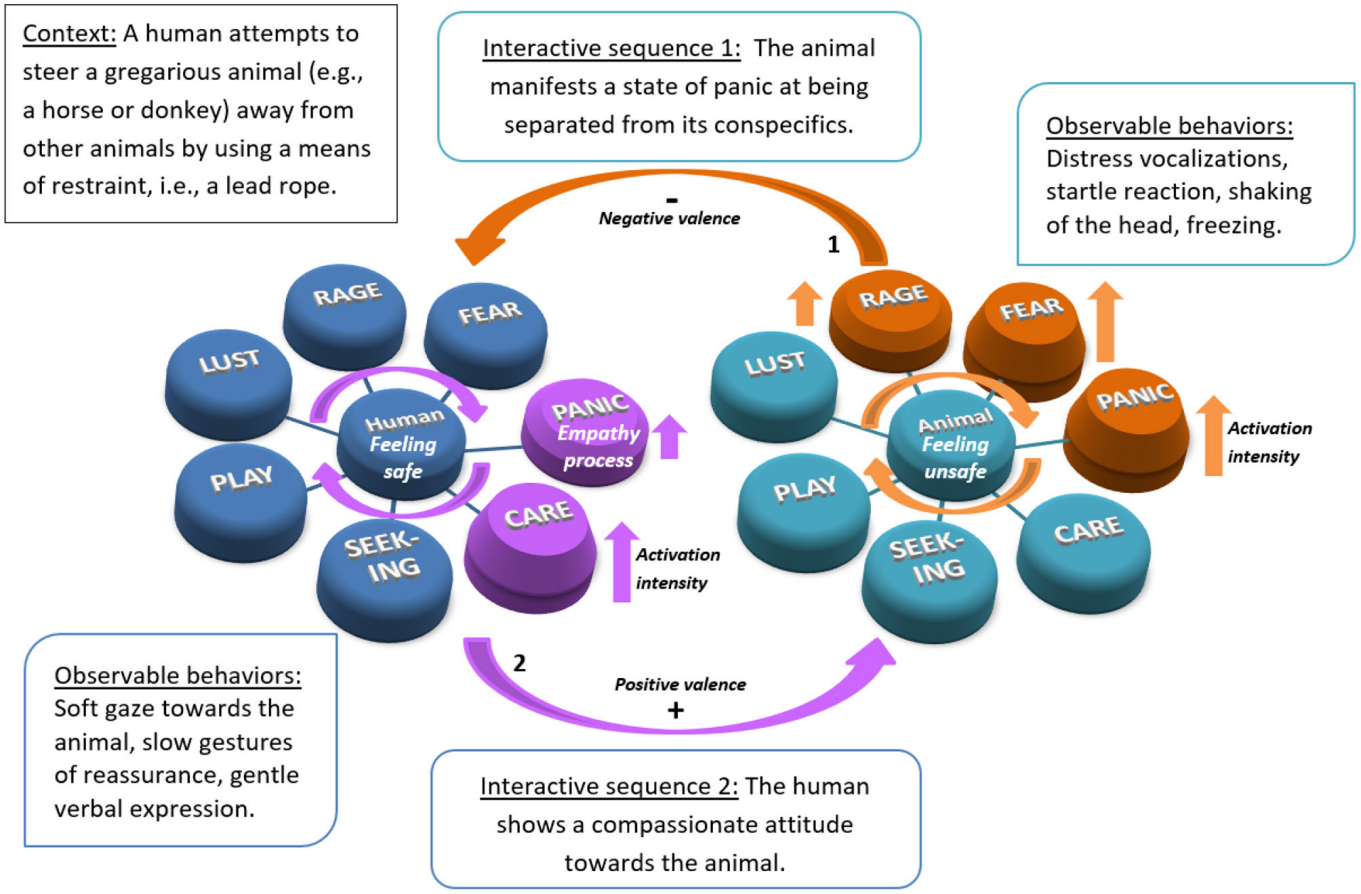
Example of hypothetical IMHAI modeling of the interspecies interactive emotional regulation process.

To describe and study the interactive sequences between humans and animals, it is first necessary to define the context in which the two individuals interact, notably by attempting to draw an inventory of the safety vs. unsafety cues (external and internal) possibly perceived by the human and the animal. Secondly, the observer notes the behaviors displayed by the two protagonists during the interaction, reporting the valence of the behaviors and mimics observed, and the effect they produce in the respective individuals in terms of neuronal, physiological and behavioral activation. We can then choose to illustrate, as in the case below in Figure 3, a particular moment of the interaction.

Figure 3 illustrates an example of a hypothetical IMHAI modeling of the interspecies interactive regulation process, integrating the notions of the activation of primary emotional systems presented in section Emotional Contagion and of emotional communication previously defined in section Toward a Systemic One Health-One Welfare Model of HAI. Here, we present a situation in which a human attempts to steer a gregarious animal, e.g., a horse or donkey, away from its conspecifics, using a restraint such as a halter and a lead rope, in order to provide care. In interactive sequence 1: the animal exhibits a state of panic at being separated from its fellow congeners; frightened, it emits distress vocalizations, calling to the others. The animal shakes its head when the human applies pressure on the halter with the lead rope. It next manifests a startle response when it begins to follow the human and then suddenly stops, no longer wanting to move forward. We hypothesize that these reactions are mediated by the PANIC, RAGE, and FEAR defensive systems respectively, with autonomic activation exerted primarily by the sympathetic nervous system (SNS). The animal perceives the situation as dangerous. In sequence 2: in response, the human finally manifests a compassionate attitude. He releases the pressure on the halter and directs his gaze toward the animal, making slow gestures in an attempt to reassure it, and addresses the animal in a gentle tone. The human perceives the situation as safe, the animal's behavior does not diminish his feeling of safety. We hypothesize that the human's behaviors are mediated by social emotional systems (PANIC and CARE systems), with autonomous regulation by the vagus nerve. A slight activation of the separation distress system (PANIC system) is associated with the process of empathy toward the animal while the activation of the CARE system is involved in the generation of benevolent and compassionate behaviors. However, positive and sustained affective bonds must be developed for the animal's PANIC system to remain stable when the human suggests that it move away from its fellow congeners in order to carry out other activities with it. In this context, when the PANIC system remains stable, it is assumed that the human is recognized as an emotionally meaningful partner by the animal.

## Application Perspectives of the IMHAI

We propose the IMHAI as a mid-level model serving as a framework for advancing various domains involving HAI. Application of the IMHAI could be extended to the following different settings:

- the modeling of dyadic interactions, such as the interactions between a companion animal and its owner or between a working animal and its owner;
- the modeling of triadic interactions, particularly in the fields of Animal-Assisted Interventions (i.e., patient-animal-therapist) or horseback riding (i.e., rider-horse-teacher);
- analysis of group dynamics generated by the activation and regulation of several primary emotional systems.

In addition, we suggest several examples of possible applications of IMHAI in the fields of HAI, HAR and HAB. With regard to interactions with companion animals, livestock, laboratory, zoo and wild animals (HAI/HAR), the IMHAI could help field professionals identify, through behavioral observation, the emotional processes present in the animal in order to anticipate certain behaviors or responses and potentially prevent accidents and injuries that could occur during interaction with the animal. The training of professionals in the use of training and learning methods based on positive reinforcement by stimulating the activation of the SEEKING exploration system as well as of social emotional systems such as the CARE and PLAY systems guarantees the improvement of working conditions and animal handling. As such, the IMHAI could serve as a basis for teaching purposes and pedagogical concept knowledge, in order to analyze the actions that had contributed to an endangerment or, conversely, led to beneficial outcomes during HAI. Given that the understanding of primary emotional systems is common to both humans and animals, the settings studied may also target the identification of emotional responses in humans which contribute to coregulation or, oppositely, to codysregulation during interspecies interactions.

In addition, application of the IMHAI could help advance the understanding of relationship building (HAR) and bonding (HAB) between humans and animals. This model can be used as a scaffold for the description of the emotional processes occurring during HAI, allowing to reflect both the valence of the interactions (+/–) and the communicational value of the emotion (translating the relationship that individual A wishes or not to establish with individual B), depending on each individual and each animal species observed. In addition, the study of interactive emotional regulation could be investigated through, for example, the measurement of the autonomic state by measuring the heart rate variability of each protagonist. These studies should help to better define the relationships between the activation of the different primary emotional systems. Hence, the study of the PANIC and CARE systems is likely to lead to promising breakthroughs for the understanding of human-animal relationships and the development of attachment bonds.

Understanding the underlying processes present during human-animal interactions has also become a fundamental issue in the field of animal-assisted interventions (AAI). For example, we plan to use the IMHAI to study “patient-animal-therapist” interactions in animal-assisted therapy. A better understanding of emotional processes during HAI should enable: (i) a better understanding of the role of the animal during the intervention in order to promote animal welfare in AAI, in particular through the promotion of mutually beneficial relationships between humans and animals, and also (ii) to more accurately pinpoint the strategies used by the therapist when taking advantage of the relationship with the animal for the purposes of care. This knowledge should ultimately enable advances in the assessment of AAIs, both in terms of effectiveness and efficiency.

## Conclusion

Recognizing that animals have emotions and consciousness (121) changes the way we perceive and view these living beings. Such recognition is the cornerstone of a better understanding of human-animal relationships and the development of attachment bonds. Through the process of empathy, we become more receptive to their needs and suffering, while seeking ways to address these needs. An emotional adjustment can then occur, promoting a change in mindset and attitudes toward animals. It is no longer a matter of dominating, coercing or conditioning, but rather of developing a genuine relationship based on trust, respect and reciprocity of exchanges. Thus, emotional communication could enable a true understanding on both sides, decreasing at-risk situations and increasing the establishment of social bonding.

The theoretical contributions integrated in the IMHAI provide a conceptual framework for the study of affect-related behavior and interspecies interactions, promoting their respective wellbeing from a One Health-One Welfare perspective. With this article, we propose a systemic approach to interspecies primary emotions that should lead, on the one hand, to a better understanding of HAI and, on the other, to foster mutually beneficial interspecies relationships. In a first step, we investigated the concept of emotional transfer with the processes of emotional contagion, embodied communication and interactive emotional regulation, in order to establish a systemic approach to primary emotions in a social context. Within the framework of a One Health–One Welfare–One Biology approach, we proposed that emotions are a central process in the study of HAI. We notably focused on the description of the seven primary emotional systems identified by Panksepp (71) and subsequently presenting a framework for observing and assessing emotional states in the individual and during HAI. Finally, from a holistic perspective, we suggest that the feeling of safety should be considered as a main factor, since it would appear to have a crucial impact on the individual's emotional and physiological state. We argue that taking this factor into account is an essential element to prevent at-risk situations in HAI but also to promote the establishment of positive links between humans and animals. However, these concepts require future empirical research to determine whether they are able to reflect the processes at work in HAI.

Given the perpetually evolving nature of scientific knowledge, the IMHAI does not correspond to a fixed theory or model. Its objective is rather to provide a theoretical basis that can serve as a reflection tool for researchers and field professionals from various fields and disciplines, by encouraging their collaboration. Similarly to an incubator, it is our hope that it will foster fruitful reflections and discussions, which may enhance existing models or inspire new methodologies and research designs. The present work aims to provide access to scientific knowledge to field professionals, who are confronted with these issues on a daily basis. As such, the IMHAI can hopefully provide professionals with a concrete and practical framework to reflect on HAI in order to prevent at-risk situations and to promote environments and interspecies interactions that are conducive to the development of positive and sustainable social bonds. Finally, based on the knowledge gathered herein and from our field observations, different hypotheses are proposed for understanding the processes involved in HAI that remain to be investigated and substantiated in future work. To this end, we also suggest that the experience of field professionals be more fully integrated into the research process and the construction of scientific theoretical frameworks for the study of HAI, thereby creating a collaborative platform that could bring together practitioners and researchers from diverse backgrounds around a common theme, namely HAI.

## Data Availability Statement

The original contributions presented in the study are included in the article/supplementary material, further inquiries can be directed to the corresponding author/s.

## Author Contributions

All authors listed have made a substantial, direct, and intellectual contribution to the work and approved it for publication.

## Conflict of Interest

The authors declare that the research was conducted in the absence of any commercial or financial relationships that could be construed as a potential conflict of interest.

## Publisher's Note

All claims expressed in this article are solely those of the authors and do not necessarily represent those of their affiliated organizations, or those of the publisher, the editors and the reviewers. Any product that may be evaluated in this article, or claim that may be made by its manufacturer, is not guaranteed or endorsed by the publisher.

## Footnotes

^1^Using deep brain stimulation (DBS), previous studies (130, 131) have demonstrated associations between different affects and neural circuits. It has thus been shown that DBS in homologous neuronal regions generate consistent and similar emotional action tendencies in all mammals. The fact that animals exhibit rewarding and punitive states (in the sense that animals “voluntarily” turn them on or off) has been interpreted as empirical evidence for emotional content in animals.

## Abbreviations

IMHAI: Integrative Model of Human-Animal Interactions.
